# Supplementary material for: Identifying determinants of readmission and death post-stroke using explainable machine learning
Source: PLoS One. 2025 Sep 18;20(9):e0332371. doi: 10.1371/journal.pone.0332371 (PMC12445469; doi:10.1371/journal.pone.0332371)
Supplement: Attachment — Submitted filename: Review 20250420.docx [file pone.0332371.s001.docx]

This study uses a rigorous analytic approach of multiple XML models, including regression-based, tree-based, and distance-based algorithms. This diversity in modeling approaches enhances the robustness of the findings. Using 10-fold cross-validation ensures that the models are tested on different subsets of the data, reducing the risk of overfitting. The study uses a combined ranking approach, incorporating Weighted Importance Scores and Frequency Counts to identify significant predictors across models. The performance of each model is assessed using standard evaluation metrics, including accuracy, area under the ROC curve (AUC), squared-error loss, logistic loss, and misclassification rate.

However, I strongly encourage the inclusion of additional information in the study to increase the interpretability and understanding of the findings. By incorporating additional information, robustness tests, and replicability measures, the study can further enhance its credibility and impact in the field of stroke research.

1. Detailed Description of Data Preprocessing:
   - The manuscript should include a more detailed description of the data preprocessing steps, such as handling missing values, normalization, and applying feature engineering techniques.
2. Model Hyperparameters:
   - Providing information on the hyperparameters used for each XML model would enhance the study's transparency and replicability.
3. Sensitivity Analysis:
   - Including a sensitivity analysis to show how changes in model parameters or the inclusion/exclusion of certain variables affect the results would strengthen the robustness of the findings.

I would also encourage the authors to discuss the weaknesses and limitations of their modeling approach and their data sources. The following areas should be addresses:

1. Sample Size and Generalizability:
   - Acknowledge the limitations related to sample size and the regional focus of the study. Discuss how these factors might affect the generalizability of the findings.
2. Class Imbalance:
   - Address the issue of class imbalance in the dataset and how it was managed. Discuss any potential impacts on model performance and the interpretation of results.
3. Model Limitations:
   - Highlight any limitations of the XML models used, such as their reliance on specific types of data or potential biases in variable selection.
4. Data Availability:
   - Mention any restrictions on data availability and how they might limit the ability of other researchers to replicate the study.

Finally, the only thing missing from this study is a complete outline of the potential implications of these findings. I would like to know how these conclusions can be used without the following areas:

1. Clinical Practice:
   - Discuss how the findings can be applied in clinical practice to improve post-stroke care, resource allocation, and patient outcomes. Highlight any specific recommendations for healthcare providers.
2. Policy and Public Health:
   - Explore the implications for public health policy, particularly in addressing socioeconomic disparities and improving care transitions for stroke survivors.
3. Future Research:
   - Identify areas for future research, such as developing interventions based on the identified predictors, validating the models in different populations, and exploring additional variables.

By including these additional details, the manuscript can provide a more comprehensive and nuanced understanding of the study's findings, their implications, and the context in which they were derived. This will also help address potential concerns and enhance the research's replicability and applicability.
