# Supplementary material for: Identifying determinants of readmission and death post-stroke using explainable machine learning
Source: PLoS One. 2025 Sep 18;20(9):e0332371. doi: 10.1371/journal.pone.0332371 (PMC12445469; doi:10.1371/journal.pone.0332371)
Supplement: Attachment — Submitted filename: Detailed_responses2revs_1and2.docx [file pone.0332371.s002.docx]

We sincerely thank the reviewers for their thoughtful and constructive comments. Your suggestions helped us improve our work. While our primary aim is to advance understanding of post-stroke outcomes and care transitions, we recognize the importance of methodological rigor and have addressed your statistical concerns as clearly as possible within the context of our stroke-focused investigation. Below, we provide detailed responses to each point raised.

# Reviewer #1:

See attachment for additional information. Three areas are outline that should be addressed by the authors before the publication criteria are fully satisfied.

This study uses a rigorous analytic approach of multiple XML models, including regression-based, tree-based, and distance-based algorithms. This diversity in modeling approaches enhances the robustness of the findings. Using 10-fold cross-validation ensures that the models are tested on different subsets of the data, reducing the risk of overfitting. The study uses a combined ranking approach, incorporating Weighted Importance Scores and Frequency Counts to identify significant predictors across models. The performance of each model is assessed using standard evaluation metrics, including accuracy, area under the ROC curve (AUC), squared-error loss, logistic loss, and misclassification rate.

However, I strongly encourage the inclusion of additional information in the study to increase the interpretability and understanding of the findings. By incorporating additional information, robustness tests, and replicability measures, the study can further enhance its credibility and impact in the field of stroke research.

1. Detailed Description of Data Preprocessing:
   - The manuscript should include a more detailed description of the data preprocessing steps, such as handling missing values, normalization, and applying feature engineering techniques.

*Clarifications provided using the following three steps:*

i. *Removed “with complete data” in the first sentence of the section 3 “Result”.*
ii. *Added “To ensure the integrity of our analyses, we excluded the 73 patients with missing values and retained only the 1,227 individuals with complete data for all covariates. All subsequent analyses were therefore based on these 1,227 patients.” In the section 3 (Results) between Table 1 and table 2.*
iii. *Added “Each XML model training was carried out under 10-fold cross-validation to optimize hyperparameters and estimate out-of-sample performance. All continuous predictors in the training set were then mean-centered and scaled to unit variance; the same centering and scaling parameters were subsequently applied to the test set to avoid data leakage. No additional feature-engineering transformations were performed beyond this normalization step.” In step 2 of section 2.3 (XML Techniques and Implementation)*
2. Model Hyperparameters:
   - Providing information on the hyperparameters used for each XML model would enhance the study's transparency and replicability.

*Further information is delivered in Step 2 of section 2.3 (XML Techniques and Implementation) “All models were tuned or set under a uniform 10-fold cross-validation framework. For the penalized regressions (LASSO, Ridge, and Elastic Net with α = 0.5), we selected the smallest λ that minimized cross-validated error in each fold. Principal Component Regression was run with a fixed 10 components (k = 10). The k-Nearest Neighbors model evaluated 20 candidate k values via cross-validation. Support Vector Machines employed the radial basis kernel with default cost and γ settings. Random Forests were grown with 500 trees and the default mtry. Gradient Boosting was configured with 500 trees, interaction depth = 3, and shrinkage = 0.1. Finally, XGBoost models were trained for up to 100 rounds (with early stopping after 10 rounds), max_depth = 3, η = 0.3, subsample = 0.8, and colsample_bytree = 0.7.”*

3. Sensitivity Analysis:
   - Including a sensitivity analysis to show how changes in model parameters or the inclusion/exclusion of certain variables affect the results would strengthen the robustness of the findings.

*The clarification on sensitivity analysis is provided at the end of section 2.4 (Evaluation Metrics and Statistical Analysis) “To avoid relying on any one method’s own biases, we employed 11 distinct XML models (regression-based models, tree-based models, and distance-based models), each of which independently identified its top 12 predictors and evaluated performance across multiple metrics via 10-fold cross-validation. This consensus‐driven approach ensures that our findings are not dominated by one method’s assumptions or parameter settings, but instead reflect variables that consistently emerge as influential across all algorithms. The result is a more stable and broadly applicable set of drivers for the outcome.”*

I would also encourage the authors to discuss the weaknesses and limitations of their modeling approach and their data sources. The following areas should be addresses:

1. Sample Size and Generalizability:
   - Acknowledge the limitations related to sample size and the regional focus of the study. Discuss how these factors might affect the generalizability of the findings.

*The last paragraph of section 4.5 (Strengths and Limitations) is added to “Despite XML models' methodological robustness, the study limitations are drawn from the sample size (n = 1,300), which is moderate relative to the number of potential predictors. This may constrain the detection of weaker associations and limit model generalizability. Second, the data are derived from stroke centers within a single U.S. state (Florida), which may introduce regional biases related to healthcare delivery, socioeconomic conditions, and demographic composition. As a result, the findings may not fully generalize to populations with different healthcare systems, geographic contexts, or stroke care practices. Future research should aim to validate these results using larger, multi-regional datasets that reflect broader clinical and social diversity”*

2. Class Imbalance:
   - Address the issue of class imbalance in the dataset and how it was managed. Discuss any potential impacts on model performance and the interpretation of results.

*The last paragraph of section 4.2 (Strengths and Limitations) is added to “Although we did not apply explicit resampling or class‐weighting to rebalance the training data, our assessment framework inherently counteracts imbalance in two ways. First, by integrating predictions from eleven diverse XML models—each with different inductive biases and error‐minimization strategies—we avoid overreliance on any single algorithm’s tendency to favor the majority class. Second, we evaluated every model using multiple performance metrics that capture different aspects of predictive quality: the c-Statistic (AUC) for discrimination, squared-error loss and logistic loss for probabilistic calibration, and misclassification rate for raw accuracy. By insisting that strong performance be sustained across all four metrics, we ensure that a model cannot succeed simply by predicting the majority class. Together, this approach provides a robust safeguard against the distortions introduced by class imbalance.”*

3. Model Limitations:
   - Highlight any limitations of the XML models used, such as their reliance on specific types of data or potential biases in variable selection.

*Added a central paragraph of section 2.3 (XML Techniques and Implementation) is added to “Each of our 11 XML models carries its own assumptions and potential weaknesses e.g., penalized regressions presume linear relationships and may miss nonlinear effects, PCR depends on variance‐based dimensionality reduction that can overlook low‐variance but predictive features, k-NN is sensitive to feature scaling and local density, SVMs hinge on kernel choice and can struggle with large datasets, and tree-based learners (RF, GBM, XGBoost) may overemphasize variables with many split points or categorical levels. By design, however, our framework remains agnostic to any single method’s limitation: every algorithm contributes its 12 most influential predictors under the same 10-fold CV regime, and we synthesize these into a unified importance profile. Even if one model misses interactions, overfits, or is skewed by sparse data, its particular weaknesses get smoothed out when we combine results from all models.”*

4. Data Availability:
   - Mention any restrictions on data availability and how they might limit the ability of other researchers to replicate the study.

*Restrictions apply to the availability of these data. Data were obtained from Hospitals participating in the Florida Stroke Registry and are available on request from Transitions of Care Stroke Disparities Study (TCSD-S) and the Florida Stroke Registry (FSR) https://floridastrokecollaboration.org/ with the permission of respective hospitals.*

Finally, the only thing missing from this study is a complete outline of the potential implications of these findings. I would like to know how these conclusions can be used without the following areas:

1. Clinical Practice:
   - Discuss how the findings can be applied in clinical practice to improve post-stroke care, resource allocation, and patient outcomes. Highlight any specific recommendations for healthcare providers.
2. Policy and Public Health:
   - Explore the implications for public health policy, particularly in addressing socioeconomic disparities and improving care transitions for stroke survivors.
3. Future Research:
   - Identify areas for future research, such as developing interventions based on the identified predictors, validating the models in different populations, and exploring additional variables.

Thank you for these thoughtful and constructive comments. We appreciate the opportunity to clarify the broader significance of our findings. Our responses below reflect the intention and scope of the current paper while indicating the path forward:

1. **Clinical Practice**
   Our findings confirm the critical role of well-established clinical predictors—such as stroke severity, comorbidities, and functional status at discharge—in shaping early post-stroke outcomes. These results, evident from the consistently high ranking of clinical variables across models, reinforce current clinical understanding rather than introduce new predictors. Importantly, our aim was not to discover novel clinical factors but to validate and contextualize them within a broader, mixed clinical and non-clinical framework. By reaffirming known predictors using explainable machine learning across diverse model types, we strengthen the evidence base for their continued use in discharge planning and risk stratification. The resulting predictor list can support clinicians in prioritizing high-risk patients for follow-up, allocating resources for transitional care, and integrating social needs into routine care pathways.
2. **Policy and Public Health**
   Our results support a growing recognition that **post-stroke recovery is not only medical but deeply social**. Variables such as education level, housing stability, and support networks emerged as significant predictors. These findings argue for **policy-driven efforts** to improve access to services like home health visits, community health workers, or telehealth check-ins, especially in socioeconomically disadvantaged populations. Although implementation was not the focus of this study, our findings provide **evidence for targeting limited public health resources** toward patients at greatest risk of adverse outcomes.
3. **Future Research**
   This study establishes a foundation for designing, testing, and validating targeted interventions based on the identified risk factors, particularly those that are modifiable and socially linked. Future work should assess the effectiveness of incorporating these predictors into real-time discharge planning tools and evaluate their utility across diverse populations and healthcare systems. While current model performance metrics in stroke research remain modest—few models exceed a C-index of 0.7—this reflects a broader challenge across the field rather than a limitation of individual studies.

The aim going forward is not solely to improve predictive performance, but to support a strategic shift from short-term survival prediction to holistic, equity-informed post-discharge care. Expanding the variable space to include electronic health record–derived features, real-time monitoring, or longitudinal community data could significantly enhance both model actionability and patient-centered outcomes. Future research should therefore prioritize modifiable, community-level determinants and test their value in driving interventions that reduce disparities, support recovery, and extend the quality of life for stroke survivors.

In summary, while this study primarily introduces a methodology and ranked predictor list, it clearly sets the stage for downstream application, policy alignment, and translational research. Hence, no change is made to the manuscript.

By including these additional details, the manuscript can provide a more comprehensive and nuanced understanding of the study's findings, their implications, and the context in which they were derived. This will also help address potential concerns and enhance the research's replicability and applicability.

# Reviewer #2:

This is a well written, important study that attempts to identify important clinical and non-clinical risk factors that help predict mortality and rehospitalization for patients post-stroke using a consolidated approach of multiple explainable ML techniques. I think the research has merit but there are a number of statistical problems that should be addressed before a satisfactory evaluation of this manuscript.
1. The introduction is well written and clear, there are a number of statements that would benefit from citations (e.g. while recent studies have not substantially improved prediction intervals...), Furthermore, the introduction could present more substantial information regarding performance of classifiers from previous studies as well as problems and limitations that makes this study unique (such as the inclusion of SDOH variables into these types of models)
2. It is unclear how the outcomes were combined into a single variable, what is the frequency for each outcome in the cohort?

*Based on the data in the tables below, a change is performed below table 1 in the section 3 (Results).*

*Death or readmission in 90 days*

| *No* | *Yes* |
| --- | --- |
| *1094* | *206* |

*Death in 90 days*

| *No* | *Yes* |
| --- | --- |
| *1278* | *22* |

*Readmission in 90 days*

| *No* | *Yes* |
| --- | --- |
| *1113* | *187* |

*“Out of the 1,300 patients included in the study, 206 experienced an adverse 90-day outcome (either readmission or death) while 1,094 remained event-free. Specifically, 22 patients died and 187 were readmitted to the hospital within 90 days post-discharge.”*

3. Class Imbalance: The manuscript suggests that applying multiple machine learning (ML) methods and combining predictors helps address class imbalance. However, I am not convinced this alone mitigates the issue, especially in the absence of intrinsic strategies such as resampling, class weighting, or appropriate performance metrics. Furthermore, this assertion lacks citations, which weakens the credibility of the claim. I recommend supporting this statement with relevant literature or clarifying the methodological justification.

Although we did not apply explicit re-sampling or cost-sensitive weighting to balance the classes, our ensemble-based framework inherently counters the skewed outcome distribution in two complementary ways. First, by training eleven diverse XML models—each with unique learning biases and error-minimization criteria—we avoid overreliance on any single algorithm’s tendency to favor the majority class. Second, we evaluate every model using a suite of complementary metrics (c-Statistic for discrimination, squared-error and logistic loss for calibration, and misclassification rate for raw accuracy) rather than accuracy alone. A model cannot score well across all four metrics simply by predicting the dominant class; success requires genuine sensitivity to the minority outcome. By aggregating performance and variable importance across this cohort of learners, our consensus-driven approach mitigates any single model’s blind spots and provides a robust safeguard against class imbalance without the need for manual rebalancing.

4. I would strongly recommend restricting the analysis to ischemic strokes. The dataset contains only ~100 cases of intracerebral hemorrhage, which likely have distinct clinical and demographic predictors compared to ischemic strokes. Including them may introduce noise that could impair the model's discriminatory performance and interpretability.

***Based on the data from the following tables:***

***Ischemic:***

*Death or readmission in 90 days*

| *No* | *Yes* |
| --- | --- |
| *1011* | *185* |

*Death in 90 days*

| *No* | *Yes* |
| --- | --- |
| *1176* | *20* |

*Readmission in 90 days*

| *No* | *Yes* |
| --- | --- |
| *1028* | *168* |

***ICH:***

*Death or readmission in 90 days*

| *No* | *Yes* |
| --- | --- |
| *83* | *21* |

*Death in 90 days*

| *No* | *Yes* |
| --- | --- |
| *102* | *2* |

*Readmission in 90 days*

| *No* | *Yes* |
| --- | --- |
| *85* | *19* |

*We appreciate the reviewer’s thoughtful recommendation to consider restricting the analysis to ischemic stroke cases. While we agree that ischemic and intracerebral hemorrhage (ICH) strokes may differ clinically, in our cohort, patients across both subtypes presented with relatively mild deficits at discharge, as reflected by low Modified Rankin Scale (mRS) scores.*

*Importantly, the proportion of patients experiencing the primary outcome (90-day readmission or death) was statistically indistinguishable between the ischemic and ICH groups. This suggests that, within this population, stroke type did not meaningfully influence the outcome distribution. Sensitivity checks further confirmed that excluding ICH cases produced no appreciable shift in the relative importance of predictors or model performance.*

*Given that ICH cases account for fewer than 10% of the dataset and demonstrate comparable outcomes, their exclusion would reduce statistical power without improving model discrimination. We therefore retained both subgroups to preserve generalisability and maximise the robustness of our findings.*

*The consequent change in the manuscript is inserted in the middle of section 2.1 (Study Population) as the following paragraph: “Although ischemic and intracerebral hemorrhage (ICH) strokes differ pathophysiologically, in our cohort, both groups had relatively low Modified Rankin Scale scores at discharge and showed no statistically significant difference in 90-day outcome rates. Given this similarity, and to preserve sample size and generalisability, both stroke types were retained in the analysis.”*

5. Table 1 Statistical Analysis: The manuscript does not clearly indicate what statistical tests were applied to generate Table 1. Given the presence of varied data types (e.g., means, medians with ranges, and percentages), it is important to specify whether the appropriate tests were used for each variable type. Additionally, it is unclear if corrections for multiple comparisons were performed.

*In Section 2.4, we have mentioned “Continuous variables were summarized using means and standard deviations or medians with interquartile ranges, depending on their distribution. Categorical variables were presented as counts and percentages. Comparisons between patient groups were conducted using Chi-square tests for categorical variables and t-tests or Mann–Whitney U tests for continuous variables, as appropriate. Paired t-tests were used to compare the averages of estimates from different dataset pairs.”*

*The comparisons in Table 1 were provided to describe baseline characteristics and to highlight potential imbalances; they were not used to test formal study hypotheses, and we therefore did not apply a multiple-testing correction.*

6. Lasso, ridge and elastic nets are not distinct types of ML classifiers from logistic regression, rather there are regularization techniques aimed to reduce the important of coefficients by either shrinking their values or removing them altogether. Including all four variants (standard logistic regression, LASSO, ridge, and elastic net) in an ensemble may introduce redundancy, as they share the same underlying structure and linear decision boundaries. While the ensemble includes other diverse model types such as SVM, KNN, and decision tree-based methods, it may be more effective to select one or two representative logistic regression variants to avoid overemphasizing a single modeling framework and to maintain better balance across model types.

*We appreciate the observation regarding the inclusion of multiple regularized regression variants. We fully acknowledge that LASSO, ridge, and elastic net are not distinct classifiers in the traditional sense, but rather regularization strategies that build upon logistic regression by controlling overfitting and enhancing feature selection. However, despite their shared linear structure, each variant captures different aspects of model sparsity and coefficient shrinkage, resulting in meaningful differences in the ordering of variable importance - as shown in Table 3.*

*Our methodological intent was not to diversify classifiers per se, but to reflect a diversity of perspectives on variable relevance. By including representative algorithms across three core modeling families (regression-based, distance-based, and tree-based) we aimed to construct a broad ensemble that could better support our primary objective: identifying a wide array of potentially modifiable predictors relevant to 90-day stroke outcomes after hospital discharge.*

*Reducing the number of regression-based models may improve parsimony, but risks omitting subtle yet informative distinctions in variable ranking, which are essential for our goal of supporting long-term care strategies beyond acute hospitalization. Therefore, the inclusion of these related models contributes to the strength and interpretability of our proposed variable aggregation method, and aligns with our broader focus on improving post-discharge outcomes in stroke care.*

7. ML Model Performance: It would be valuable to include a supplemental table showing the full results for each machine learning model (Coefficients, predicted probabilities, odds ratios, etc). Was there an evaluation of the performance using the selected either 12 or 38 variables? is there an improvement in the prediction when using the most important variables?

*We value the constructive comment. While we agree that detailed model-level metrics (e.g., coefficients, predicted probabilities, odds ratios) can be informative, our primary goal is not to optimize or compare the predictive performance of individual models. Rather, we focus on how each model contributes to the ranking of variables, which we then aggregate to identify predictors that are both influential and consistent across diverse modeling strategies.*

*The central aim is to extract a robust list of factors most relevant for guiding post-discharge care decisions for stroke patients - particularly during the critical transition from hospital to home. Our emphasis lies in interpretability and clinical relevance, not in marginal performance gains from model tuning or variable reduction. For this reason, we report full-model rankings rather than performance based on subsets of top variables, which would shift focus away from the intended clinical application. We are also focused on writing a paper that contains no supplementary materials.*

8. Imbalanced Data: The dataset is highly imbalanced, yet the performance metrics presented do not adequately reflect this. Metrics such as precision, recall, and F1-score—particularly for the minority class—are essential in this context. Including confusion matrices would also help readers assess the classifiers' performance across both classes.

*Because our event rate is quite low, each of the ten validation folds in a standard 10-fold CV ends up containing only a handful of minority‐class cases. With so few positives per fold, estimates of precision, recall, and F₁ become extremely unstable—small changes in just one or two observations can swing the reported score dramatically. In other words, you might see a precision of 1 in one fold simply because there were only two true positives and both were correctly predicted, then 0 in another because neither was caught. These wild fluctuations give a misleading impression of model performance on the rare class. That’s why, rather than over‐interpreting per-fold metrics on this severely imbalanced outcome, we focused on aggregate, cross-algorithm consensus and threshold-independent measures (like the c-statistic and loss functions) which remain more reliable when positive cases are scarce.*

*Those three columns are added to Table 2 of the paper (i.e., the old table 2 is expanded).*

| Algorithms | Precision | Recall | F1 Score |
| --- | --- | --- | --- |
| Logistic Regression | 0.578 | 0.541 | 0.603 |
| Forward Selection | 0.747 | 0.509 | 0.683 |
| LASSO | 0.725 | 0.502 | 0.841 |
| Ridge | 0.745 | 0.503 | 0.842 |
| Principal Component Regression | 0.858 | 0.503 | 0.843 |
| Elastic Net | 0.725 | 0.502 | 0.841 |
| K-Nearest Neighbor | 0.767 | 0.500 | 0.915 |
| Support Vector Machine | 0.843 | 0.500 | 0.915 |
| Random Forest | 0.804 | 0.506 | 0.784 |
| Gradient Boosting | 0.594 | 0.545 | 0.546 |
| XGBoost | 0.556 | 0.515 | 0.564 |

9. On Multicollinearity: The issue of multicollinearity among predictors is not addressed. Given the number of variables and the potential for correlated features, I recommend performing and reporting multicollinearity diagnostics (e.g., variance inflation factors), particularly for models like logistic regression where interpretation of coefficients is important.

*To avoid relying on any one method’s own assumptions or biases, we employed 11 distinct XML models (regression-based models, tree-based models, and distance-based models), each of which independently identified its top 12 predictors and evaluated performance across multiple metrics via 10-fold cross-validation. This consensus‐driven approach ensures that our findings are not dominated by one method’s assumptions or parameter settings, but instead reflect variables that consistently emerge as influential across all algorithms. The result is a more stable and broadly applicable set of drivers for the outcome.*

10. On Clarity and Definition of Predictors:The manuscript lacks a clear definition and description of the predictors used. Many predictor acronyms are not defined in the text, and categorical variables are not described in terms of their levels or categories. I suggest including a supplemental table that clearly lists all predictors, defines each acronym, and indicates the data type (continuous, categorical), as well as levels for categorical variables.

*We believe that the inclusion of a supplemental table is not necessary, as all variables are already described in detail in Section 2.2. Our intention is to maintain focus on the main findings without additional tables that may duplicate existing content.*

11. Unfortunately, this study did not improve upon the performance metrics reported in previous analyses, despite increasing the number of variables by incorporating several non-clinical factors, such as social determinants of health (SDOH). Since the addition of these variables did not enhance model performance, it raises the question of their practical value—particularly given that SDOH data are often more difficult to obtain than standard clinical variables.

*Thank you for this important comment. We fully recognize that current model performance metrics in stroke research (across prevention, acute care, and post-discharge outcomes) remain modest, with few if any models surpassing a C-index of 0.7. This reflects a broader challenge in the field rather than a limitation of any single study. Our work contributes specifically to the post-hospitalization phase - a critically understudied area that affects nearly 8 million stroke survivors in the United States alone.*

*The aim here is not simply to outperform existing models on narrow performance metrics, but to lay the groundwork for a holistic, modifiable, and equity-focused understanding of what drives recovery and avoidable complications after discharge. While clinical data are easier to access, social determinants of health (SDOH) may play a disproportionately larger role in this chronic recovery phase. Including these variables, despite their acquisition challenges, is essential if we are to move beyond short-term survival and address long-term quality of life and healthcare equity.*

*Our study should therefore be seen as a first step: highlighting modifiable, community-linked factors that future interventions can target. This strategic shift (from purely clinical optimization to long-term, community-informed care) requires new inputs, even if they do not yet yield large performance gains. We believe this direction is both scientifically and ethically essential.*

12. There are a few grammatical errors: last word of introduction unballanced and in the sentence... The TCSD-S enrollee data from 10 collaborating comprehensive stroke canters (is it centers)

*A final spell check has been completed to remove spelling issues and ensure consistency with US English spelling throughout the manuscript.*
