# Supplementary material for: Identifying determinants of readmission and death post-stroke using explainable machine learning
Source: PLoS One. 2025 Sep 18;20(9):e0332371. doi: 10.1371/journal.pone.0332371 (PMC12445469; doi:10.1371/journal.pone.0332371)
Supplement: Attachment — Submitted filename: Review 20250612.docx [file pone.0332371.s003.docx]

This study uses explainable machine learning (XML) to identify clinical and non-clinical predictors of 90-day readmission and mortality among stroke survivors. Drawing on data from the *Transitions of Care Stroke Disparities Study* (TCSD-S), the authors analyzed outcomes for 1,300 patients using 11 machine learning models. The study integrates information from clinical registries, neighborhood-level social determinants of health (SDOH), and transition-of-care metrics. The key contribution is a composite list of 38 variables, derived from model consensus, that predict adverse post-stroke outcomes. The authors argue for the superiority of XML over traditional regression methods, highlighting the added value of SDOH in predictive modeling and the potential application in digital health systems.

**Contribution of the Methodology, Insights, Conclusions:**

1) The manuscript extends current clinical studies by integrating socioeconomic, neighborhood, and behavioral characteristics into the machine learning framework to enhance model relevance.
2) The authors clearly describe model selection, performance metrics, and variable importance aggregation (frequency count and weighted score), providing transparency and reproducibility.
3) The application of 11 XML models to identify consensus predictors is a novel and methodologically rigorous approach to multidimensional health outcome modeling that is strengthened by the 10-fold cross-validation and inclusion of multiple performance metrics (C-statistic, log loss, precision, etc.).
4) These findings could inform risk-stratification tools and targeted post-discharge interventions.

**Additional Clarity Needed Before Publication**

1) **Outcome Construction:** The composite outcome of “90-day readmission or mortality” is not adequately justified. The frequency of each outcome (readmission vs. death) is provided late in the paper (n=22 deaths, n=187 readmissions), suggesting imbalanced outcome events. Provide a more explicit rationale for combining these outcomes or present separate models for each. At minimum, sensitivity analyses using each outcome separately would enhance interpretability.
2) **Additional Preprocessing Details:** While the authors briefly mention normalization and the exclusion of 73 individuals due to missing data, there is insufficient detail on how missing values were handled or imputed. It would be beneficial if the authors explicitly described missing data patterns, imputation methods (if used), and any variable selection criteria. A flowchart could help illustrate data preprocessing.
3) **Limited Generalizability:** The sample is geographically confined to Florida, and 92% of strokes are ischemic. Only ~100 ICH cases are included, limiting broader applicability. Please consider performing a secondary analysis that is restricted to ischemic stroke only or provide stratified results. Discuss generalizability limitations more prominently in the abstract and conclusion.
4) **Imbalance:** The adverse outcome rate is relatively low (15.8%), yet the authors do not apply formal balancing techniques (e.g., SMOTE, class weights). It would be beneficial if the authors included an additional discussion on how class imbalance may or may not influence metrics such as precision and recall.
5) **Explainability:** The manuscript emphasizes “explainable” ML but does not attempt to interpret the most influential variables beyond summary tables. I suggest including a short narrative interpretation of how top predictors could inform clinical decision-making.
