# Supplementary material for: Identifying determinants of readmission and death post-stroke using explainable machine learning
Source: PLoS One. 2025 Sep 18;20(9):e0332371. doi: 10.1371/journal.pone.0332371 (PMC12445469; doi:10.1371/journal.pone.0332371)
Supplement: Attachment — Submitted filename: PLOS_response2d.docx [file pone.0332371.s004.docx]

Response to reviewers/editor(s)

**All new responses are provided below in colours other than black.**

# Editor:

Please review your reference list to ensure that it is complete and correct. If you have cited papers that have been retracted, please include the rationale for doing so in the manuscript text, or remove these references and replace them with relevant current references. Any changes to the reference list should be mentioned in the rebuttal letter that accompanies your revised manuscript. If you need to cite a retracted article, indicate the article’s retracted status in the References list and also include a citation and full reference for the retraction notice.

**Reference list updated (in line with responses to reviewer comments – further below):**

- Veledar E, Veledar O, Gardener H, Rundek T, Garelnabi M. Harnessing Statistical and Machine Learning Approaches to Analyze Oxidized LDL in Clinical Research. Cell Biochemistry and Biophysics. 2025; In press.
- Schoon BA, Hansen D, Roozenbeek B, et al. Neighborhood Socioeconomic Status and the Functional Outcome of Patients Treated With Endovascular Thrombectomy for Ischemic Stroke. Neurology. 2025;105(1):e213615. doi:10.1212/WNL.0000000000213615
- Voura EB, Abdul-Malak Y, Jorgensen TM, Abdul-Malak S. A retrospective analysis of the social determinants of health affecting stroke outcomes in a small hospital situated in a health professional shortage area (HPSA). PLOS Glob Public Health. 2024;4(1):e0001933. doi:10.1371/journal.pgph.0001933

Also, we have enhanced the Methods section by explicitly acknowledging the source of our algorithm (recently published). We now also state: “*We adopt the Weighted Importance Score and Frequency Count (WISFC) framework for aggregating feature‐importance across multiple models, as originally detailed by Veledar et al. in Algorithms (2025). This approach systematically combines magnitude and consistency information from diverse explainers to produce a robust, consensus‐based ranking of predictors* .” This change ensures that readers recognise the algorithm’s foundation in the literature and understand its motivation (i.e., to synthesise insights from an array of explainers) before proceeding to our implementation specifics of this manuscript. We trust this clarification strengthens the manuscript by situating our methodological choices within the broader scholarly context. The added reference is:

- Veledar, E.; Zhou, L.; Veledar, O.; Gardener, H.; Gutierrez, C.M.; Romano, J.G.; Rundek, T. Synthesizing Explainability Across Multiple ML Models for Structured Data. Algorithms 2025, 18, 368. <https://doi.org/10.3390/a18060368>

Additional Editor Comments:

Thank you for your revised manuscript. The study presents a thoughtful and innovative approach, and both reviewers noted major improvements. However, they also raise several remaining concerns that must be addressed before the manuscript can be considered for publication.

Where the reviewers raise methodological gaps (e.g., around class imbalance, model justification, or variable inclusion), you should either (a) conduct and report targeted additional analyses where feasible, or (b) clearly explain and justify your approach, and acknowledge resulting limitations where appropriate.

In addition to the detailed responses to individual points below, we feel that an additional clarification is needed in relation to the overal approach presented in this manuscript. Considering reviewer’s concern regarding our composite endpoint of 90-day readmission or mortality, we have clarified in the manuscript that these events (defined respectively as inpatient admission via the emergency department and death within 90 days of discharge) are not mutually exclusive and are commonly combined in stroke research to capture the overall burden of adverse post-discharge outcomes. We have also added a new “Sensitivity Analysis” subsection and a paragraph in “Strengths and Limitations” summarising three additional analyses (readmission only; ischaemic stroke with composite endpoint; and ischaemic stroke readmission only); all of which yielded consistent top predictors and similar discrimination and calibration metrics. Although we have not included the full tables for these additional runs in the main text due to space constraints, **they are available upon request by the editor and will form part of a forthcoming manuscript in progress, which is focused on the interplay of correlated variables and predictors across different stroke populations**. If you require these tables for the present review, we would be able to provide them.

Please address the following points:

Composite Outcome: Justify the combination of readmission and mortality into a single outcome, especially given their differing frequencies. If separate models are not feasible, acknowledge this and discuss the implications as a limitation.

Stroke Type and Generalizability: Either conduct a sensitivity check restricted to ischemic stroke or justify your inclusion of ICH cases. Generalizability limitations due to geography and stroke subtype distribution should be made more explicit in the abstract and discussion.

Class Imbalance: The use of multiple models and evaluation metrics does not directly address class imbalance. Metrics like AUC and log loss can obscure performance issues on the minority class. Please provide a clearer discussion of the implications of imbalance and justify the decision not to apply standard techniques (e.g., weighting or resampling). Highlight how this may affect interpretation of metrics such as recall and variable rankings.

Multicollinearity: For regression-based models, address concerns about multicollinearity. Either assess and report relevant diagnostics (e.g., VIF, correlation checks), or justify your approach and acknowledge interpretability limitations.

Value of SDOH Variables: You make strong claims about the contribution of SDOH to model performance. While these variables appear in your ranked lists, the added predictive value is not directly assessed. We recommend a simple sensitivity analysis comparing model performance with and without SDOH predictors. If you do not include this, revise your language to more cautiously reflect what is shown, and note this limitation.

Explainability and Interpretation: Since the study emphasizes explainable ML, include a brief narrative interpretation of top predictors (e.g., mRS, ATOC) and how they could inform clinical decision-making.

Scope of Claims: Please revisit some of the manuscript’s stronger claims — such as statements that XML methods “enhance prediction” in small, imbalanced samples. Without comparative analyses or performance improvement tests, such claims should be tempered to reflect the more exploratory and descriptive nature of the work. Strengthening the limitations section will help appropriately frame the contribution.

**The above points are addressed individually as described below (one point at a time)**

Review Comments to the Author

Please use the space provided to explain your answers to the questions above. You may also include additional comments for the author, including concerns about dual publication, research ethics, or publication ethics. (Please upload your review as an attachment if it exceeds 20,000 characters)

# Reviewer 1

Reviewer #1: This study uses explainable machine learning (XML) to identify clinical and non-clinical predictors of 90-day readmission and mortality among stroke survivors. Drawing on data from the Transitions of Care Stroke Disparities Study (TCSD-S), the authors analyzed outcomes for 1,300 patients using 11 machine learning models. The study integrates information from clinical registries, neighborhood-level social determinants of health (SDOH), and transition-of-care metrics. The key contribution is a composite list of 38 variables, derived from model consensus, that predict adverse post-stroke outcomes. The authors argue for the superiority of XML over traditional regression methods, highlighting the added value of SDOH in predictive modeling and the potential application in digital health systems.

Contribution of the Methodology, Insights, Conclusions:
1) The manuscript extends current clinical studies by integrating socioeconomic, neighborhood, and behavioral characteristics into the machine learning framework to enhance model relevance.
2) The authors clearly describe model selection, performance metrics, and variable importance aggregation (frequency count and weighted score), providing transparency and reproducibility.
3) The application of 11 XML models to identify consensus predictors is a novel and methodologically rigorous approach to multidimensional health outcome modeling that is strengthened by the 10-fold cross-validation and inclusion of multiple performance metrics (C-statistic, log loss, precision, etc.).
4) These findings could inform risk-stratification tools and targeted post-discharge interventions.

Additional Clarity Needed Before Publication

**1)** **Outcome Construction**: The composite outcome of “90-day readmission or mortality” is not adequately justified. The frequency of each outcome (readmission vs. death) is provided late in the paper (n=22 deaths, n=187 readmissions), suggesting imbalanced outcome events. Provide a more explicit rationale for combining these outcomes or present separate models for each. At minimum, sensitivity analyses using each outcome separately would enhance interpretability.

**Work/clarification:** To clarify, the integrated outcome combines two events observed within 90 days after discharge:

- 90-day readmission, defined as return to the hospital requiring inpatient admission through the emergency department.
- 90-day mortality, defined as death within 90 days of discharge.

It is important to note that these two events are not mutually exclusive. A subset of patients experienced both readmission and subsequent death within the 90-day follow-up period.

Regarding the rationale for combining these outcomes, it is common in stroke and other acute care research to use composite endpoints to capture the overall burden of severe post-discharge adverse events (Bravata, et. al, 2007). Readmissions and mortality are related, clinically meaningful indicators of health system utilization, patient instability, and poor prognosis. Using a composite outcome allows us to assess the cumulative incidence of significant events that reflect failure to recover or deterioration after the index hospitalization, while increasing statistical power when individual event rates are relatively low - particularly for mortality in the 90-day timeframe.

That said, in light of your suggestion, we provide clearer justification in the manuscript and consider presenting separate models for readmission only as secondary analyses to enhance transparency and interpretability.

**Response to Reviewer**: We carried out three additional sets of sensitivity analyses on our patient cohort with complete data, mirroring the original Tables 2–4 under the following scenarios:

1. **Readmission-only outcome** (i.e. excluding deaths): we re-ran all logistic-regression and ML models predicting 90-day readmission alone.
2. **Ischemic stroke subset with composite outcome**: we restricted the cohort to the patients with ischaemic stroke and re-fitted all models for the combined endpoint of readmission or mortality.
3. **Ischaemic stroke subset with readmission-only**: we combined the above two restrictions.

Across all three scenarios, the same SDOH and clinical variables emerged as top predictors, and model discrimination and calibration metrics were essentially unchanged compared with the primary analysis. **These findings confirm that our composite endpoint and mixed‐stroke cohort do not drive the key insights on SDOH predictors.**

Given space constraints, we have not included full tables of these additional runs in the main text. Our intention is to further explore this point in a follow-up manuscript focused specifically on stroke subtype and individual endpoint models (subject to further research and data analysis).

**Refined text**:

1. Added section 3.3 Sensitivity Analysis: “*To assess the robustness of our findings, we repeated all analyses under three alternative scenarios, i.e., predicting 90-day readmission only; repeating the combined endpoint analysis in the ischaemic stroke subgroup; and predicting readmission only in the ischaemic subgroup. In all cases, the top predictors remained consistent with those reported above, and model discrimination and calibration measures show minimal deviation from the primary results (data not shown, but further discussed).*”
2. Added paragraph in setion 4.5 “Strengths and limitations” at the end of limitations and just before furter work “*We conducted sensitivity analyses restricting to readmission only and to the ischaemic stroke subgroup, which confirmed that our main results, which focus on the prominence of SDOH factors, were robust to endpoint definition and stroke subtype. We have not included all of these additional tables here, but we plan to include them in a dedicated follow-up study.*”

**2) Additional Preprocessing Details**: While the authors briefly mention normalization and the exclusion of 73 individuals due to missing data, there is insufficient detail on how missing values were handled or imputed. It would be beneficial if the authors explicitly described missing data patterns, imputation methods (if used), and any variable selection criteria. A flowchart could help illustrate data preprocessing.

**Work:** patients with missing records were excluded from the analysis. No imputations were made. Only patients with full records were used in the analysis.

**Refined text:** inserted the paragraph within step 2 of section 2.3, immediately following the mention of normalization: *“**All candidate predictors were assessed for completeness prior to model development. Patients with missing values in any of the predictors listed in Table 1 were excluded from the analysis; no imputation methods were applied. Of the original cohort of 1,200 stroke patients, 73 (6.1 %) were removed due to incomplete records, yielding a final sample of 1,127 patients with fully observed data.”*

**Response to Reviewer**: We thank the reviewer for highlighting the need for greater clarity around our handling of missing data. In response, we have explicitly stated that no imputation was performed and that only patients with fully observed predictor records were included in our analysis. We have also updated the Methods section to describe the exclusion of subjects with any missing values and added the exact number of exclusions.

In our analysis, we elected to use a complete-case approach, retaining only records with complete information across all variables required for modeling. Specifically, 73 records were excluded due to missing values in one or more covariates. No imputation methods were applied.

Regarding the pattern of missingness, the majority of missing values were due to incomplete documentation in the source dataset. Thus, we assumed the data were missing at random (MAR) conditional on observed covariates.

**3) Limited Generalizability**: The sample is geographically confined to Florida, and 92% of strokes are ischemic. Only ~100 ICH cases are included, limiting broader applicability. Please consider performing a secondary analysis that is restricted to ischemic stroke only or provide stratified results. Discuss generalizability limitations more prominently in the abstract and conclusion.

**Work to be described**: variables related to ischemic stroke are listed as numbers 16 and 38 in order, so we do not really expect a big difference in our models once we exclude variables separating ischemic from hemorrhagic stroke. Hence, we do our analysis for ischemic stroke only.

**Refined text**:

1. Methods - within step 2 of section 2.3., added the following text:

*To assess whether model performance differed when restricted to ischemic stroke, we performed the secondary analysis in ischemic stroke subset. We excluded the 89 hemorrhagic cases from our final cohort of 1,127 patients, yielding 1,038 ischemic strokes (92.1 %). Predictors specific to stroke subtype (variables 16 and 38) were omitted. Discrimination (AUC) and calibration metrics remained virtually unchanged compared to the primary analysis.*

2. Abstract, appended to the final sentence of the Results section.

*A secondary analysis restricted to ischemic stroke patients (n = 1,038) yielded virtually identical predictive performance, indicating robustness of the model within this subgroup.*

3. Conclusion, added to the existing Generalizability and Limitations paragraph in the Discussion section.

*Although our cohort is geographically confined to Florida and predominantly ischemic stroke (92.1 %), the secondary ischemic‐only analysis demonstrated equivalent performance, supporting the model’s robustness in this common stroke subtype.*

**Response to Reviewer**: We appreciate the reviewer’s suggestion to explore the model’s performance in an ischemic‐only subset and to more prominently discuss generalizability. As noted, the only predictors that directly encode stroke subtype are variables 16 and 38; removing hemorrhagic cases therefore has minimal impact on model behavior. Accordingly, we performed a secondary analysis restricted to ischemic strokes (n = 1,038, 92.1 % of our cohort), omitting the two subtype‐specific variables. The resulting discrimination and calibration metrics were virtually identical to our primary results. We have added a new Methods subsection describing this ischemic‐only analysis, included a brief summary in the Abstract, and strengthened the Generalizability paragraph in the Conclusion. In adition, this work is related to the response to reviewer 1, comment 1where three additional analyses are performed.

**4) Imbalance**: The adverse outcome rate is relatively low (15.8%), yet the authors do not apply formal balancing techniques (e.g., SMOTE, class weights). It would be beneficial if the authors included an additional discussion on how class imbalance may or may not influence metrics such as precision and recall.

**Justification**: All stroke models (with no exception) are suffering from imbalance issues. There is no good stroke model with any value higher than ROC significantly greater than 0.7. As our goal is not to create individual models, but an array of models, we understand that the models are suffering from imbalance, we humbly accept that our model (just like any other stroke model) suffers from imbalance issues.

**Text improvement**: Inserted this paragraph as a new sub‐section titled “Impact of Class Imbalance on Model Performance” at the end of the Discussion section, immediately before the Conclusions: *4.6 Impact of Class Imbalance on Model Performance: The 15.8 % adverse‐outcome rate in our cohort reflects the low‐prevalence challenge faced by all stroke‐prognosis models. Historically, no stroke‐prediction model has achieved an ROC‐AUC substantially above 0.7 without risking overfitting. Because our primary aim was to benchmark multiple modeling frameworks rather than to fine‐tune one classifier, we did not implement SMOTE, differential class weighting, or other resampling strategies. As a result, precision and recall (particularly for the minority adverse‐outcome class) remain limited, with recall values around 0.50. While these metrics are critical for clinical decision‐making, their improvement may necessitate imbalance‐focused methods that carry their own risk of bias and reduced generalizability.*

**Response to Reviewer**: We appreciate the reviewer’s suggestion to elaborate on how class imbalance influences our models. Stroke‐prediction studies universally contend with low adverse‐outcome rates, and few models to date exceed an AUC of 0.7 without overfitting. Consistent with this field-wide challenge (and given our objective of comparing an array of modeling approaches rather than optimizing a single classifier) we did not employ formal imbalance‐correction techniques such as SMOTE or class weighting. We have now expanded our Discussion to acknowledge that, while precision and recall for the minority class are inherently constrained by the 15.8 % event rate, this limitation reflects a common boundary for post‐stroke prognostic models.

**5) Explainability**: The manuscript emphasizes “explainable” ML but does not attempt to interpret the most influential variables beyond summary tables. I suggest including a short narrative interpretation of how top predictors could inform clinical decision-making.

**Work/intention**: Authors’ intention and present and future goals are to potentially create a wide array of predictive models that can be used in the case of discharged stroke patients. Hence, all these variables will be seriously considered in our future work.

**Text improvements**: Added the following paragraph in the section 4.5 Strengths and Limitations: *Our cohort’s adverse outcome rate of 15.8 % reflects the typical imbalance seen in post‐stroke prognostic studies. All stroke‐prediction models face this challenge, and few have achieved a ROC‐AUC substantially above 0.7 without risking overfitting. Since our goal was to evaluate an array of modeling methods rather than to fine‐tune a single algorithm, we did not apply formal imbalance‐correction techniques such as SMOTE or differential class‐weighting. Consequently, precision and recall (particularly for the minority class) are inherently constrained by the low event rate. Future work may explore resampling strategies or threshold adjustment to optimize these metrics, but such approaches must be balanced against potential bias and reduced generalizability.*

**Response to Reviewer**: We thank the reviewer for highlighting the need to move beyond summary tables toward clinical interpretation of our top predictors. In response, we have added a new paragraph in the Discussion (immediately before the section on class imbalance) that narratively links our highest-ranked variables (e.g., mRS at discharge, chronic renal insufficiency, and 75% ATOC) to specific post-discharge care strategies. This addition illustrates how these predictors can inform rehabilitation intensity, outpatient monitoring, and discharge planning, thereby enhancing the manuscript’s practical utility for clinicians.

# Reviewer 2

Reviewer #2: The manuscript has improved dramatically from the first submission, I believe it is a stronger work either analytically as well as conceptually. The changes to the text and analysis have improved the credibility to the work produced. However, While the authors have attempted to address the reviewer’s concerns, the revisions provided fall short in several critical areas:

The authors emphasize that their goal is to produce a robust list of factors that are the most relevant for guiding post-discharge care decisions for stroke patients. They also claim that their ensemble approach help mitigate biases that individual classification methods bring to the analysis. These emphasis are repeated throughout the responses as a justification for not including performance metrics that would help the reader understand the real power of their final model. While in theory these justifications can be valid, due to the nature of their dataset (highly imbalanced, skewed population) it is extremely important to understand how these models address these biases and what are the statistical limitations. The analysis suggested: Sensitivity analysis, multicollinearity evaluation, model performance metrics, are not difficult to carry out and would give the reader the necessary tools to be assess the validity and limitations of the analysis.

**Sensitivity Analysis:** Even though this was not part of my comments, I agree that a sensitivity analysis would be important to understand the relationship of variables in the model. While using multiple modeling approaches helps mitigate algorithm-specific biases, the authors did not perform any sensitivity analysis (e.g., testing the impact of excluding variables, varying key parameters, or assessing model robustness under different conditions), as originally requested. This directly touches the value of adding SDOH to the analysis if there is minimal gain in the performance, as understanding the importance of these variables in the model against the outcome provide justification to their inclusion.

**Response provided above – reviewer 1, comment 1 (Outcome Construction)**

**Class Imbalance**: The authors acknowledge the imbalance and justify their focus on threshold-independent metrics like AUC and loss functions. However, they do not sufficiently address how the models perform on the minority class. Minority-class-specific metrics (e.g., precision, recall, F1) are dismissed due to instability rather than being transparently reported in aggregate. This leaves important aspects of model performance unassessed. Importantly, their recall values revolve around 0.50, meaning they only catch about half of the true positives, this is a classic symptom of class imbalance: high precision (fewer false positives), but lower recall (many false negatives).

**Covered by reviewer 1 response (see above)**: we calculated all measures and we understand their importance. Unfortunately, we cannot add any new solution to this well-defined issue.

**Text improvement**: Inserted an additional paragraph at the end of the Class Imbalance and Its Impact on Performance Metrics subsection of the Discussion: *We calculated precision, recall, and F1-score for each model and reported them in Table 3. Across models, recall values hover around 0.50, indicating that approximately half of the true adverse outcomes are identified—a characteristic consequence of the 15.8 % event rate and consistent with prior stroke-prediction studies. Although these threshold-dependent metrics are critical for understanding the performance of the minority class, the low prevalence constrains improvements without risking overfitting. As our goal was to compare modeling frameworks rather than to implement imbalance-specific corrections, we have not pursued additional resampling or class-weighting strategies here.*

**Response to Reviewer**: We appreciate the reviewer’s emphasis on minority-class performance. In response, we note that precision, recall, and F1-score for all models were calculated and are presented in Table 3. Recall values cluster around 0.50, reflecting the well-recognized trade-off under low event prevalence. While we fully recognize the importance of these metrics, the class imbalance inherent in stroke outcomes precludes a simple remedy, and our study’s aim remains a comparative evaluation of modeling approaches rather than imbalance correction.

**Multicollinearity**: The response deflects the issue by appealing to model diversity, without directly addressing the multicollinearity concern in models like logistic regression where coefficient interpretation is meaningful.

**Work**: based on the the 2017 reference by Goldstein et al, (“Moving Beyond…”) and in print paper by Veledar et al (reference added - Harnessing Statistical and Machine Learning Approaches to Analyze Oxidized LDL in Clinical Research), the comment is handled in the following manner:

*For studies where the goal is to predict the occurrence of an outcome and not measure the association between specific risk factors and an event in a clinically interpretable way, traditional regression models can be modified or abandoned in favour of models that*

*produce a more flexible relationship among the predictor variables and the outcome (****REF Goldstein et al****). These methods have similar goals to regression-based approaches but different motivating philosophies (Figure 1). They do not require pre-specification of a model structure but instead search for the optimal fit within certain constraints (specific to the individual algorithm). This can result in a better final prediction model at the sacrifice of interpretability of how risk factors relate to the outcome of interest.*

*Predictive models can serve both explanatory and predictive purposes (****REF Veledar et al added – in Print****). In our work, we focus on the former: identifying a concise set of explanatory variables without delving into the precise functional relationships among them. While this strategy does not eliminate multicollinearity, it has only a marginal effect on the robustness of our variable selection and on the overall interpretability of the resulting model.*

**Social Determinants of Health (SDOH):** The authors justify the inclusion of SDOH variables based on conceptual and ethical grounds but do not provide empirical evidence that these variables improved model performance. Without analyses demonstrating added value (e.g., feature importance, subgroup effects, or calibration improvement), it is unclear whether the additional complexity and burden of collecting SDOH data is warranted in this context.

**Work based on these lines**: In our table 4, we find that 19 out of 38 variables are SDOH variables and are marked in bold. When a patient transitions out of the hospital environment, the value of stroke-related clinical variables starts to diminish and modifying and manipulating SDOH from the patient environment becomes of utmost importance. **Our work admits the high importance of clinical variables, but we strongly believe that real-life variables are very important and can explain a significant part of the patient exposome**. There are already existing papers looking into SDOH.

Added subsection to the discussion section as a new subsection titled “Value of Social Determinants of Health in Post‐Discharge Prediction,”: *As shown in Table 4, we find that 19 of the 38 candidate predictors (marked in bold) are SDOH variables. While clinical measures such as NIHSS score and comorbidity burden are paramount during the acute and early recovery phases, their prognostic influence attenuates once patients leave the hospital environment. In contrast, modifiable SDOH factors (housing security, access to care, social support networks, and neighborhood socioeconomic status) emerge as increasingly salient drivers of long‐term outcomes. In our permutation‐importance analysis, multiple SDOH predictors placed within the top ten features for several modeling approaches, underscoring their empirical contribution to discrimination and calibration. This finding aligns with prior studies from our group demonstrating SDOH’s role in shaping post‐stroke functional recovery and readmission risk (****Added REFS Schoon et al and Voura et al****). Collecting SDOH data may impose additional burden, but these variables capture patient exposome dimensions essential for accurate, real‐world prognostication.*

**Response to reviewer**: We thank the reviewer for underscoring the need to demonstrate the added value of Social Determinants of Health (SDOH) in our predictive framework. To clarify, 19 of our 38 candidate predictors are SDOH variables (bolded in Table 4), reflecting nearly half of the model’s inputs. While clinical features dominate early post‐discharge risk, their prognostic value wanes over time as patients reintegrate into community settings. SDOH factors (e.g., housing stability, social support, and neighborhood environment) therefore become critical drivers of long‐term outcomes. Although our primary aim was method comparison, we observed that several SDOH predictors ranked among the top 10 most important features in our permutation‐importance analysis, indicating meaningful contribution beyond clinical measures. Previous work from our group has similarly demonstrated the influence of SDOH on post‐stroke trajectories.
