# Supplementary material for: Identifying determinants of readmission and death post-stroke using explainable machine learning
Source: PLoS One. 2025 Sep 18;20(9):e0332371. doi: 10.1371/journal.pone.0332371 (PMC12445469; doi:10.1371/journal.pone.0332371)
Supplement: Attachment — Submitted filename: ResponseToEditor and Reviewers.docx [file pone.0332371.s005.docx]

Dear Editor,

Thank you for coordinating the thoughtful reviews and for providing us with the opportunity to strengthen our manuscript. We have carefully considered each comment and revised the text—most notably renaming Section 4.6, adding a targeted narrative on socioeconomic predictors, and tempering claims around model superiority—to better reflect the scope and limitations of our work.

**Response to comment 1:**

Thank you for this suggestion. We have updated the manuscript accordingly:

a. Section Renaming: Section 4.6 has been renamed to “May Modeling Help Post-Acute Stroke Care” Because that question is really still very open.
b. New Narrative Paragraph: We added a concise narrative in Section 4.6 that links our highest-ranked predictors (particularly the 19 socioeconomic factors) to specific clinical actions, such as tailoring discharge planning and coordinating home-based or community services. This highlights how explainable insights can inform post-acute care interventions rather than merely ranking variable importance.

We trust this will strengthen the paper’s focus on real-world applicability of our explainable modeling framework.

**Response to comment 2:**

We have softened our original claim about model superiority and reframed the sentence to highlight the exploratory nature of our multi-model approach and the critical role of environmental variables (including SDOH). The previous statement has been replaced with a new paragraph, which emphasizes that our goal is to leverage machine learning for preliminary insights in a domain that currently lacks robust theories or interpretability methods for post-acute stroke outcomes, rather than to assert the unequivocal superiority of any single model.

Best Regards,

Emir Veledar
